# Constraints on Letter-in-String Identification in Peripheral Vision: Effects of Number of Flankers and Deployment of Attention

**DOI:** 10.3389/fpsyg.2013.00119

**Published:** 2013-03-13

**Authors:** Myriam Chanceaux, Jonathan Grainger

**Affiliations:** ^1^Laboratoire de Psychologie Cognitive, Centre National de la Recherche Scientifique, Aix-Marseille UniversityUMR 7290, Marseille, France

**Keywords:** letter perception, crowding, non-adjacent flankers, number of flankers, spatial attention

## Abstract

Effects of non-adjacent flanking elements on crowding of letter stimuli were examined in experiments manipulating the number of flanking elements and the deployment of spatial attention. To this end, identification accuracy of single letters was compared with identification of letter targets surrounded by two, four, or six flanking elements placed symmetrically left and right of the target. Target stimuli were presented left or right of a central fixation, and appeared either unilaterally or with an equivalent number of characters in the contralateral visual field (bilateral presentation). Experiment 1A tested letter targets with random letter flankers, and Experiments 1B and 2 tested letter targets with Xs as flanking stimuli. The results revealed a number of flankers effect that extended beyond standard two-flanker crowding. Flanker interference was stronger with random letter flankers compared with homogeneous Xs, and performance was systematically better under unilateral presentation conditions compared with bilateral presentation. Furthermore, the difference between the zero-flanker and two-flanker conditions was significantly greater under bilateral presentation, whereas the difference between two-flankers and four-flankers did not differ across unilateral and bilateral presentation. The complete pattern of results can be captured by the independent contributions of excessive feature integration and deployment of spatial attention to letter-in-string visibility.

## Introduction

What factors influence our ability to identify a single letter presented in a random string of letters? Answering this question is important because it will help develop our understanding of the very first phase of the reading process during which visual feature information is mapped in parallel onto position-coded letter identities in both central and peripheral vision (Grainger and van Heuven, 2003; Marzouki et al., 2013). The importance of understanding the mechanisms involved has been nicely illustrated by the recent finding that increased letter spacing can facilitate reading in dyslexic children (Perea et al., 2012; Zorzi et al., 2012). Indeed, crowding is one factor known to have a large influence on letter-in-string perception, and manipulating inter-letter spacing modulates the amount of crowding. Crowding, or lateral masking, is the phenomenon whereby target identification in peripheral vision is affected by the presence of nearby flanking elements (Bouma, 1970; Pelli et al., 2004; Pelli and Tillman, 2008). In the present study we focus on one specific aspect of crowding effects – the contribution of non-adjacent flanking elements – while examining possible interactions between crowding effects and the deployment of spatial attention.

### Crowding and non-adjacent flankers

Effects of lateral interference on letter identification is a well-studied phenomenon in experimental psychology, with resultsshowing that target letters flanked by other letters to the left and to the right, are harder to identify than isolated letter targets (e.g., Bouma, 1970; Huckauf and Nazir, 2007; Grainger et al., 2010). More recently, these effects of lateral interference have been integrated within the wider perspective of crowding effects on the processing of various kinds of visual stimuli (e.g., Pelli et al., 2004; Pelli and Tillman, 2008; see Levi, 2008; Whitney and Levi, 2011, for reviews). However, the vast majority of these studies have limited their investigation to the effects of stimuli that are the closest to the target, and have shown that target-flanker separation determines target identification accuracy. Much of this work can be summarized by Bouma’s law (Bouma, 1970; Pelli and Tillman, 2008), which states that critical spacing (i.e., the target-flanker separation that allows target identification at a criterion level of accuracy) is a linear function of target eccentricity. On the other hand, we currently know much less about how distractor stimuli that are not adjacent to the target, that is, are separated from the target by at least one other flanking element, might influence target identification. Yet natural cluttered environments in general, and printed text in particular, nearly always involve situations where there are non-adjacent flanking elements.

A handful of studies have tested for effects of non-adjacent flankers on letter identification in peripheral vision (Butler and Currie, 1986; Heller et al., 1995; Huckauf and Heller, 2002a,b)^1^. These studies compared the effects of two- vs. four-flankers on target letters located at the center of three-letter and five-letter strings that were presented left or right of a central fixation. The results of these studies replicated the standard finding of a drop in performance with two flanking stimuli compared with isolated targets (i.e., standard crowding), and demonstrated a further drop in performance from tow-flankers to four-flankers. A similar effect of number of flankers was also reported for digit identification by Strasburger et al. (1991).

These relatively understudied effects of non-adjacent flanking stimuli are all the more interesting in that the non-adjacent flanking stimuli typically fall outside of the critical spacing limits that determine standard crowding effects according to Bouma’s law. This has led a number of researchers to propose that different mechanisms might be involved in non-adjacent flanking effects compared with standard crowding (i.e., effects of adjacent flankers). Whereas standard crowding would mostly reflect excessive feature integration (e.g., Pelli et al., 2004; Levi, 2008; Levi and Carney, 2009), interference from non-adjacent flankers would mainly reflect an increase in positional uncertainty with longer strings (e.g., Butler and Currie, 1986; Huckauf and Heller, 2002a). In other words, processing of target identity might not be harmed so much by non-adjacent flankers, but performance would drop because of loss of information about target position. Given that all prior research has used simple identification paradigms with either full or partial report, positional uncertainty would induce incorrect responses by participants reporting the identity of an item at the incorrect position. In the present study we test for effects of number of flankers while controlling for positional uncertainty by using a two-alternative forced-choice (2AFC) procedure in which the alternative choice was not present in the display. That is, the alternative presented along with the target in 2AFC was never a letter present in the stimulus on that trial. If positional uncertainty is the key mechanism driving prior observations of a number of flankers effect, then we ought to see a much reduced effect of this manipulation in the present study.

### Effects of deployment of attention

It is also possible that effects of non-adjacent flankers reflect differences in the deployment of spatial attention as a function of string length. Strasburger et al. (1991) suggested that it might be harder to focus attention on the target location in longer strings, and this causes a cost in the processing of target identity as well as target position. This attentional account of effects of non-adjacent flankers could be integrated within a general explanation of crowding according to which spatial attention determines the size of the crowding zone (e.g., Strasburger et al., 1991; Intriligator and Cavanagh, 2001; Strasburger, 2005). According to these accounts it is possible to equate the empirically defined crowding zone (i.e., critical spacing) with the size of the spotlight of spatial attention. In line with this proposal, there is some evidence that crowding effects can be reduced by spatial cueing. This was shown most clearly by Yeshurun and Rashal (2010), who demonstrated a reduction in critical spacing for orientation identification (target was a rotated T) with the prior presentation of a spatial cue to target location. Spatial cueing has also been shown to affect the different impact of inward vs. outward flankers as a function of whether or not attention is drawn toward the fovea or not (Petrov and Meleshkevich, 2011). Furthermore, Scolari et al. (2007) reported reduced critical spacing when targets were presented in a different color to flankers, presumably because the different color helped attract attention to the target^2^.

**Figure 1:**
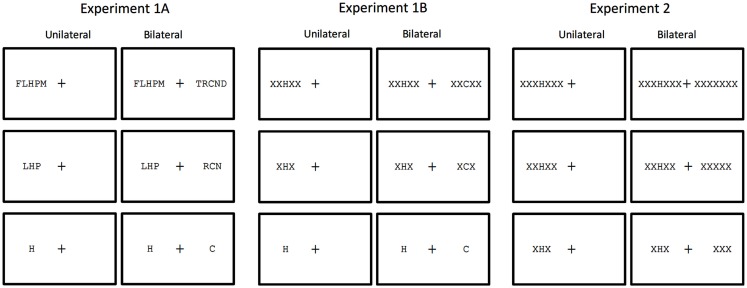
**Summary of the conditions tested in the present study in which number of flankers (zero, two, four, six) and unilateral vs. bilateral presentation were manipulated**. In all examples the target is the letter “H” presented in the LVF. Flankers in Experiment 1 are other letters. Flankers in Experiments 1A and 2 are Xs. Note that the conditions tested in Experiment 2 are the same as Experiment 1B, except that all stimuli in the contralateral field of the bilateral condition are Xs, and with the zero-flanker condition replaced by a six-flanker condition.

In order to provide a further test of attentional accounts of crowding driven by adjacent and non-adjacent flankers, we included two manipulations expected to modify the deployment of spatial attention. First, we compared flanking effects induced by random letter strings (Experiment 1A) with effects induced by homogeneous strings of Xs (Experiment 1B). The homogeneous flankers are expected to facilitate focusing of attention on the target as the odd man out (see Figure 1), and therefore to reduce flanker interference compared with non-homogeneous flankers (e.g., Scolari et al., 2007). Second, we presented stimuli either unilaterally (i.e., all stimuli grouped in one visual field) or bilaterally, with an equivalent number of stimuli presented simultaneously in the contralateral field (see Figure 1). Unilateral stimuli will enable rapid allocation of attention to target location (targets were always at the central position of strings), whereas bilateral stimuli will encourage a division of attention across the visual fields until processing of the post-cue that indicates the target location. According to attentional accounts of crowding, we should see reduced flanker interference under unilateral presentation conditions. More specifically, the combined manipulation of attentional deployment and number of flankers will allow us to examine whether adjacent and non-adjacent flankers are differentially affected by spatial attention. To do so, we plan to test for partial interactions between a given attentional manipulation and the effects of adjacent flankers (zero- vs. two-flankers) on the one hand, and the effects of non-adjacent flankers on the other.

## Experiment 1

Experiment 1 compared identification of isolated letter targets, and targets embedded as the central letter in three-letter, and five-letter strings presented in the left and right visual field under either unilateral or bilateral presentation conditions. In Experiment 1A, flanking stimuli were other letters, and in Experiment 1B flankers were homogeneous Xs (see Figure 1).

### Method

#### Participants

Students from the University of Provence were paid 5 € for their participation, 9 in Experiment 1A (8 women, mean age 22 years) and 17 in Experiment 1B (11 women, mean age 23.7 years). All participants were native speakers of French, and reported having normal or corrected to normal vision.

#### Design and stimuli

The stimuli consisted of 15 consonant letters presented in uppercase (B, C, D, F, G, H, J, K, L, M, N, P, R, S, and T). Three factors were manipulated as within-participants variables: visual field (left or right of fixation), number of flankers (zero, two, or four), and laterality (unilateral or bilateral presentation). All 15 target letters were tested twice in each of the 12 experimental conditions, leading to 360 trials (15 × 12 × 2)/participant. In Experiment 1A different consonants from the set of 15 were used as flankers in the conditions with flankers, and different combinations of flankers were used for the different targets. In Experiment 1B all flankers were the uppercase letter X. In the bilateral presentation condition, the same combination of flankers was used as in the unilateral condition for flankers in the same visual field as the target, and a different set of letters were shown in the contralateral field. Stimuli were presented in random order with all experimental conditions mixed in a single block. The task was 2AFC, thus each target letter was paired with another letter from the set of 15 consonants, and that was not present in the stimulus display (target and alternative were presented after stimulus presentation). Each of the 15 letters served as the alternative choice on the same number of trials, and were distributed equally across the different conditions. All stimuli were presented in white 21-point Courier New font on a black background.

#### Apparatus

An EyeLink 1000 eye tracker (SR Research Ltd.) was used to control for eye position. The eye tracker had a sampling rate of 1000 Hz and used an automatic saccadic detection algorithm based on a velocity threshold of 30°/s and an acceleration threshold of 8000°/s^2^, which corresponds to the cognitive configuration for the EyeLink 1000. Stimuli were presented on a ViewSonic P227 monitor with a refresh rate of 100 Hz and a screen size of 1024 × 768 pixels. Stimulus presentation was controlled with Experiment Builder software (SR Research Ltd.). Eye movements were recorded from the right eye.

#### Procedure

**Figure 2:**
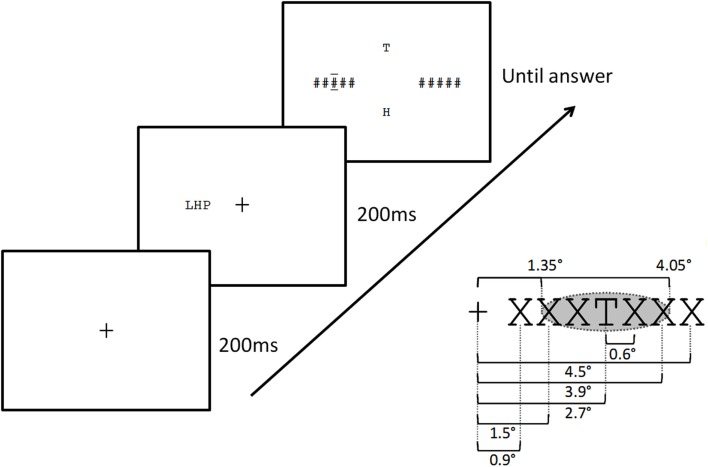
**Sequence of events on a trial in the present study (top left), and distances used (bottom right)**. Fixation cross for 200 ms, followed by the stimulus display (target and any accompanying letters) for 200 ms, followed by a post-mask accompanied by two horizontal bars placed above and below the target location, plus the presentation of two-alternative letters for the 2AFC response. Inter-letter spacing, eccentricity of the closest letter and the central target letter, and theoretical extent of the crowding zone according to Bouma’s law (shaded ellipse) are shown bottom left.

Participants were seated and asked to adjust a chinrest so that their eyes were level with the center of the computer screen. For the calibration phase, participants were asked to fixate on dots presented at nine different points on the computer screen. The calibration was repeated after a familiarization phase and then every 30 trials. The sequence of events on a trial was as follows (see Figure 2). First, participants were asked to gaze on the fixation cross that was presented at the center of the computer screen. Participants’ fixation had to first stabilize on the cross for 200 ms. If the gaze moved during more than 50 ms outside of an area of 50 × 120 pixels around the fixation cross (0.7° right or left), the cross remained on the screen. After 200 ms of continuous fixation, the target and flanker stimuli were presented for 200 ms. Stimuli were presented on one or both sides of the center of the screen aligned horizontally. Participants were seated at approximately 80 cm from the monitor. At this distance a letter subtended about 0.6° in horizontal extent, and the center of the target was at an eccentricity of 2.7°. The inter-letter spacing (center to center) was 0.6°, such that the center of the most distant flankers in the four-flanker condition were located 3.9° from fixation, at the limit of the theoretical extent of the target’s crowding zone (2.7° + 2.7°/2 = 4.05°; see Figure 2).

During stimulus presentation eye fixation had to stay on the center of the screen, otherwise the trial was canceled. After stimulus presentation, a post-mask appeared on both sides of the fixation cross, along with the two-alternative letter responses placed above and below the center of the screen. The mask consisted of two strings of five hash marks and remained on the screen until participants responded. The post-mask was accompanied by a post-cue to indicate which letter to identify, using two horizontal bars above and below one of the hash marks. The target letter was always the central letter of the string. Participants were instructed to respond as accurately as possible by pressing either the upward arrow key (for the alternative above) or the downward arrow key (for the alternative below) to indicate which letter had appeared at the cued location. Participants had to respond on each trial even if they were unsure of the answer (forced-choice). An audio tone signaled a correct response. After participants’ response a blank screen appeared and the next trial began. The experiment lasted approximately 30 min.

### Results

#### Experiment 1A: different letter flankers

**Figure 3:**
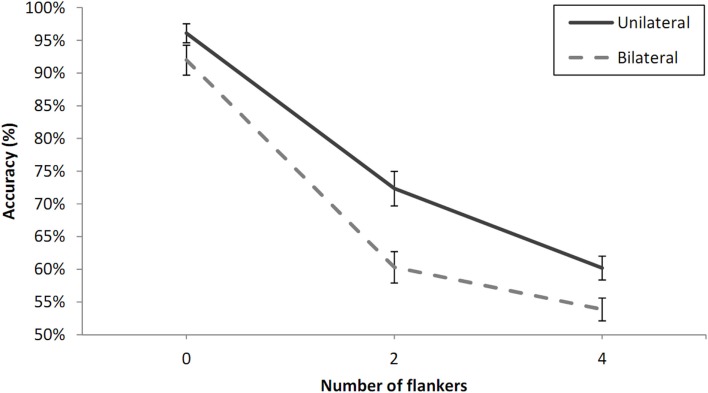
**Percent correct 2AFC to letter targets under unilateral and bilateral presentation conditions and as a function of the number of flankers in Experiment 1A**. Error bars are standard errors.

Percentage correct values averaged across participants for each experimental condition are shown in Figure 3. We checked individual accuracy values to determine that all participants were above chance-level using a chi-square test. A repeated measures analysis of variance (ANOVA) was conducted on the mean percentage correct/participant and condition. Number of flankers (zero, two, four), laterality (unilateral vs. bilateral presentation), and visual field (RVF vs. LVF) were within-participants variables. There was a clear effect of laterality with an advantage for unilateral presentation, *F*(1, 8) = 51, *p* < 0.001, and a strong main effect of number of flankers, *F*(2, 16) = 149.86, *p* < 0.001, but no main effect of visual field, *F*(1, 8) = 1.92, *p* = 0.20, and no significant interactions with this factor (Number of flankers × Visual field, *p* = 0.06, Laterality × Visual field, *p* = 0.89). The main effect of number of flankers was driven mostly by a large decrease in performance from zero- to two-flankers, *t*(8) = 18.54, *p* < 0.001, but the difference between the two- and four-flanker condition was also significant, *t*(8) = 3.9, *p* < 0.01.There was a significant interaction between number of flankers and laterality, *F*(2, 16) = 4.72, *p* < 0.05. The partial interaction between number of flankers and laterality was significant for the zero-flanker and two-flanker conditions, *F*(1, 8) = 11.37, *p* < 0.01, with stronger effects of adjacent flankers in the bilateral condition (31.7%) compared with the unilateral condition (23.8%). On the other hand, the interaction was only marginally significant when limited to the two-flanker and four-flanker conditions, *F*(1, 8) = 5.04, *p* = 0.06, and was in the opposite direction.

#### Experiment 1B: homogeneous × flankers

**Figure 4:**
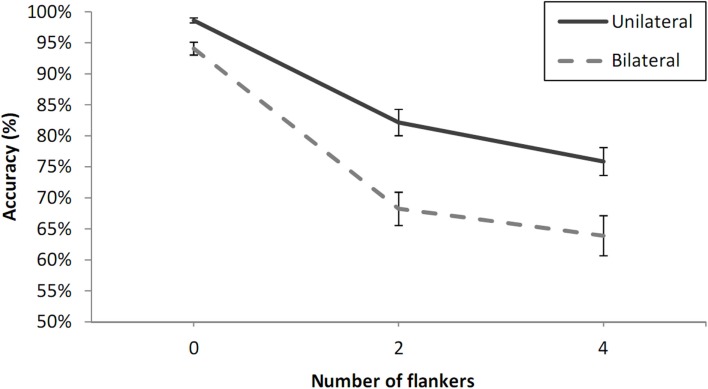
**Percent correct 2AFC to letter targets under unilateral and bilateral presentation conditions and as a function of the number of flankers (Xs) in Experiment 1B**. Error bars are standard errors.

One of the participants was excluded from further analysis because of chance-level performance according to a Chi-square test (at 0.05 level). The data from the remaining participants were analyzed in a three-way by participants analysis of variance (ANOVA) with visual field (LVF or RVF), number of flankers (zero, two, or four), and laterality (unilateral or bilateral presentation). Percentage correct values averaged across these participants for each experimental condition are shown in Figure 4. The analysis revealed main effects of number of flankers, *F*(2, 30) = 83.73, *p* < 0.001, and laterality, *F*(1, 15) = 97.97, *p* < 0.001, but no effect of visual field, *F*(1, 15) = 0.07, *p* = 0.79. As in Experiment 1A, the main effect of number of flankers was driven essentially by a large decrease in performance from zero- to two-flankers, *t*(15) = 10.16, *p* < 0.001, but the difference between the two- and four-flanker condition was also significant, *t*(15) = 3.37, *p* < 0.01. There was a significant interaction between number of flankers and laterality, *F*(2, 30) = 6.94, *p* < 0.01, with greater flanker interference under bilateral presentation. Again, this interaction was driven mostly by the zero-flanker and two-flanker conditions, for which the partial interaction between number of flankers and laterality was significant, *F*(1, 15) = 12.59, *p* < 0.01, but was not significant when examining only the two-flanker and four-flanker conditions, *F*(1, 15) = 0.54, *p* = 0.47.

#### Combined analysis of Experiments 1A and 1B

**Figure 5:**
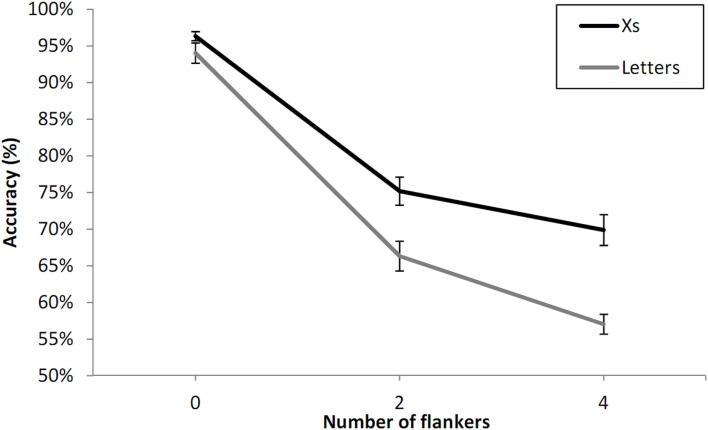
**Percent correct 2AFC to letter targets with letter flankers compared with X flankers as a function of the number of flankers in Experiment 1, and averaging across unilateral and bilateral presentation and visual field**. Error bars are standard errors.

In this joint analysis we examined the effect of type of flanker (letters vs. Xs) and the effect of number of flankers (see Figure 5). We found a significant interaction between type of flanker and number of flankers, *F*(2, 46) = 5.06, *p* < 0.05. Flanker effects were greater when the flankers were letters (Experiment 1A) than when they were Xs (Experiment 1B). The partial interaction between number of flankers and type of flanker was significant for adjacent flankers (zero- vs. two-flankers), *F*(1, 23) = 4.73, *p* < 0.05, but was not significant for non-adjacent flankers (two- vs. four-flankers), *F*(1, 23) = 2.10, *p* = 0.16.

### Discussion

Experiment 1 revealed an effect of the number of flanking letters on letter-in-string identification, in conditions where effects of positional uncertainty were minimized by always presenting targets at the center of strings, and using 2AFC. The decrement in performance from no flankers to two-flankers replicates the standard effects of crowding on letter perception (e.g., Bouma, 1970; Huckauf and Nazir, 2007; Grainger et al., 2010). The further decrement in performance from two-flankers to four-flankers replicates prior observations of an influence of non-adjacent flankers (Butler and Currie, 1986; Strasburger et al., 1991; Heller et al., 1995; Huckauf and Heller, 2002a,b). These effects of non-adjacent flankers observed with the particular paradigm used in the present study helps rule out explanations of such effects as being due to an increase in positional uncertainty.

Effects of number of flankers interacted with the presence or not of a letter string in the contralateral visual field. The two-flanker condition showed significantly stronger interference compared with the zero-flanker condition under bilateral presentation conditions. This is in line with attentional accounts of crowding (Strasburger et al., 1991; Intriligator and Cavanagh, 2001; Strasburger, 2005) according to which attentional deployment determines the extent of the crowding zone. However, contrary to this account, the difference between the two-flanker and four-flanker conditions actually diminished, albeit non-significantly, in the bilateral condition, rather than increasing. Nevertheless, this could be due to floor effects affecting performance in the bilateral four-flanker condition of Experiment 1 (see Figures 3 and 4). The same pattern was found with the type of flanker manipulation (letters vs. Xs), which significantly modulated the effects of adjacent flankers (see Figure 5), while the difference between the two-flanker and four-flanker conditions was not significantly affected by the type of flanker. Again, floor effects in the four-flanker condition could be driving this pattern. Experiment 2 therefore attempts to remove these floor effects in order to test whether crowding is systematically greater under bilateral presentation as the number of flankers increases, as predicted by attentional accounts of crowding in general, and attentional accounts of effects of non-adjacent flankers in particular.

Furthermore, in Experiment 1 there was a general advantage for unilateral presentation compared with bilateral presentation, most likely because unilateral presentation favors rapid allocation of attention toward the target location, whereas bilateral presentation favors divided attention across the visual fields (e.g., Chakravarthi and Cavanagh, 2009). In Experiment 1 the contralateral display always contained a distractor letter in the central position that was not the letter X, and this implies that participants had to process the post-cue in order to know where the target was. Experiment 2 examines to what extent the presence of a distractor letter in the contralateral field determined the deployment of attention under bilateral presentation. To do so, flankers were homogeneous Xs with no letter (other than Xs) in the contralateral field in bilateral presentation conditions (see Figure 1).

Finally, in Experiment 1 the maximum number of contiguous flankers was four elements, with two to each side of the target. Given the stimulus size and spacing and the viewing distance employed in the present study, the most eccentric flankers in the four-flanker condition were at the limits of the critical spacing as defined by Bouma’s law. It is therefore possible that the extra interference generated by the four-flanker condition compared with the two-flanker condition could be driven by the mechanisms underlying standard crowding. Experiment 2 therefore tests for effects of number of flankers but this time including a six-flanker condition replacing the no flanker condition. In this condition, the center of the most distant flankers were at 4.5° eccentricity, and hence fell outside of the crowding zone as defined by Bouma’s law (see Figure 2).

## Experiment 2

### Method

#### Participants

Twenty-two students (6 men and 16 women, mean age 22.9 years) from the University of Provence volunteered to participate. All participants were native speakers of French and reported having normal or corrected to normal vision.

#### Design and stimuli

The design and stimuli were the same as in Experiment 1, with the exception that all distractors in both unilateral and bilateral conditions were the letter X, and the number of flankers was two, four, or six. Thus, visual field (LVF or RVF) was crossed with Number of Flankers (two, four, or six) and Laterality (bilateral or unilateral) in a 2 × 3 × 2 design. Each letter was the target in 24 trials, leading to 360 trials (15 × 24).

#### Apparatus and procedure

These were the same as in the previous experiments.

### Results

**Figure 6:**
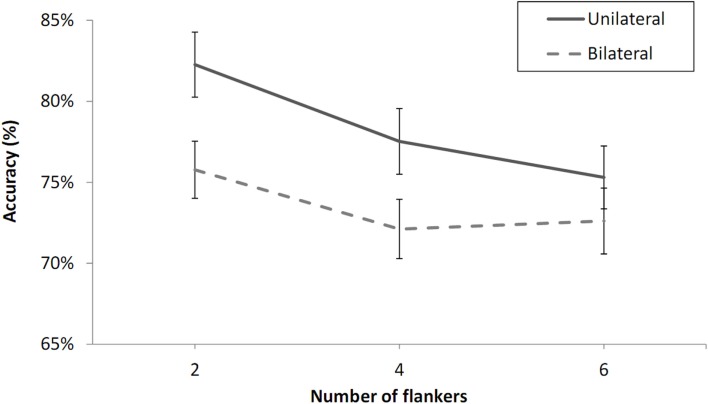
**Percent correct 2AFC to letter targets with X flankers under unilateral and bilateral presentation conditions as a function of the number of flankers in Experiment 2**. Error bars are standard errors. Note the change in scale of the *Y*-axis given the reduced effect sizes in Experiment 2 due to the absence of a zero-flanker condition.

Two of the participants were excluded from further analysis because of chance-level performance according to a Chi-square test (at 0.05 level). The data from the remaining participants (shown in Figure 6) were analyzed in a three-way by participants’ analysis of variance (ANOVA) with visual field (LVF or RVF), number of flankers (two, four, or six), and laterality (unilateral or bilateral presentation) as variables. The analysis revealed main effects of number of flankers, *F*(2, 38) = 6.28, *p* < 0.01, and laterality, *F*(1, 19) = 15.50, *p* < 0.001, but no effect of visual field, *F*(1, 19) = 0.14, *p* = 0.72. There was a significant difference between the two-flanker and four-flanker conditions, *F*(1, 19) = 7.10, *p* < 0.05, and no significant difference between the four-flanker and six-flanker conditions, *F*(1, 19) = 0.38, *p* = 0.54. Critically, there was no interaction between number of flankers and laterality, *F*(2, 38) = 1.77, *p* = 0.47.

### Discussion

The results of Experiment 2 confirmed the significant decrement in performance with four-flankers compared with two-flankers seen in Experiment 1, while revealing no significant difference between the four-flanker and six-flanker condition. This strongly suggests that effects of number of flankers, manipulated here along the horizontal meridian, are at least partly determined by the extent of the crowding zone as determined by Bouma’s law.

Furthermore, the results of Experiment 2 revealed a significant advantage for unilateral over bilateral presentation on letter-in-string identification in peripheral vision, in conditions that arguably optimize target localization prior to presentation of the post-cue, in both the bilateral and unilateral conditions. When contrasted with the results obtained in the bilateral (two- and four-flanker) conditions of Experiment 1B, the results of Experiment 2 confirm that the presence of a distractor letter in the contralateral field of the bilateral presentation condition in Experiment 1B was inducing a processing cost. This can be best attributed to the fact that in these conditions there was no cue to target location prior to presentation of the post-cue. This is akin to having two precues for target location as in the Chakravarthi and Cavanagh (2009) study. The relevant conditions in that study were the “Attend 1” and “Attend 2-Bilateral” conditions in an experiment where participants has to determine the orientation of a target letter T among a circular array of distractor Ts of varying orientation. Target location was indicated by a post-cue. In the “Attend 1” condition, target location was also indicated by a precue, whereas in the “Attend 2-Bilateral” condition, target location was precued along with another location in the opposite hemifield of the circular display. Chakravarthi and Cavanagh reported an 11% difference in performance between the single precue and dual bilateral precue conditions in their Experiment 1A, which is in line with the 12% difference found in Experiment 1B of the present study.

Finally, there was no interaction between number of flankers and laterality in Experiment 2, in conditions where there was no evidence that floor effects were affecting performance. Figure 6 shows very clearly that the difference between the two-flanker and four-flanker conditions was almost identical under unilateral and bilateral presentation conditions. This strongly suggests that the effects of non-adjacent flankers were operating independently of spatial attention in the present study.

## General Discussion

In the present study participants had to identify target letters that appeared at a post-cued location under brief stimulus presentations and pattern masking. Target letters could appear in isolation or as the central letter of three-letter, five-letter, or seven-letter strings, and the flanking elements could either be composed of a set of different letters (Experiment 1A) or a homogenous string of Xs (Experiments 1B and 2). Target and flankers were presented in peripheral vision, to the left or to the right of a central fixation. Furthermore, the target and flanking stimuli could be presented unilaterally, or bilaterally, with an equivalent number of letters in the contralateral visual field. The main findings of the present study can be summarized as follows: (1) number of flanking stimuli continued to affect letter identification beyond the two-flanker conditions of standard crowding experiments; (2) homogeneous × flankers facilitated letter identification compared with different letter flankers; (3) unilateral presentation facilitated letter identification compared with bilateral presentation; and (4) the interfering effects of adjacent flankers (zero- vs. two-flankers) were reduced under unilateral compared with bilateral presentation and with X flankers compared with different letter flankers, whereas differences between the two-flanker and four-flanker conditions were not affected by these manipulations.

The complete pattern of results reported in the present experiments can be captured by the combined influence of flanking elements and spatial attention. In what follows we discuss possible mechanisms underlying the influence of each of these factors, and their possible interactions.

### Effects of number of flankers

The results of the present study confirm prior observations of an effect of non-adjacent flanking stimuli in letter identification (Butler and Currie, 1986; Heller et al., 1995; Huckauf and Heller, 2002a,b) and digit identification (Strasburger et al., 1991). Since all this prior work has used identification paradigms with full or partial report, performance was driven by a combination of how well information about target identity and target position is processed. This led to the general consensus that the effects of non-adjacent flanking stimuli would be mostly due to positional uncertainty increasing as a function of string length. This was thought to contrast with the effects of adjacent flankers driven mainly by excessive feature integration (Pelli et al., 2004; Levi, 2008), since non-adjacent flankers typically fall outside of the target’s crowding zone (determined by Bouma’s law) and therefore cannot generate interference in this way^3^.

The use of a 2AFC procedure in the present study was designed to limit possible effects of positional uncertainty, since the alternative choice was never present in the display. Therefore, efficiency in the processing of target identity is considered to be the main factor driving performance in the present experiments, and this efficiency can be affected by flanker interference and deployment of spatial attention. Here we discuss possible mechanisms underlying flanker interference effects, and in particular the number of flankers effect found in the present study, before turning to discuss possible relations between crowding and spatial attention.

**Figure 7:**
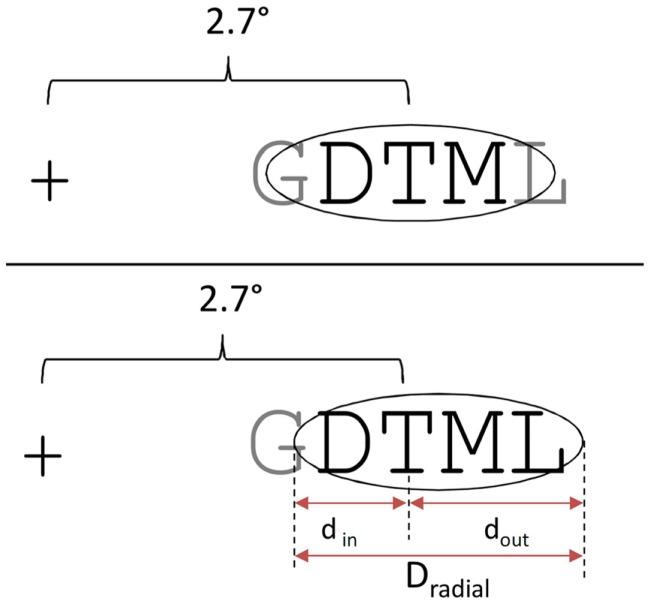
**Crowding zone determined by Bouma’s law without inward-outward asymmetry (upper panel) and with inward-outward asymmetry (bottom panel), following the model proposed by Nandy and Tjan (2012)**. This demonstrates that the inward-outward asymmetry enables a complete non-adjacent letter to fall in the crowding zone in the four-flanker condition of the present study.

One key result of the present study is that adding two additional flankers to the standard two-flanker condition, such that targets have two-flankers on either side, causes a significant decrease in target identification, whereas performance was not further affected by adding two additional flankers (i.e., the six-flanker condition tested in Experiment 2). Given the target eccentricity tested in the present study (see Figure 2), it would appear that Bouma’s law provides a good approximation of the extent of crowding observed with multiple aligned flanking elements. Interference increases as more flankers are added, as long as these flankers fall with the crowding zone defined by Bouma’s law. The fact that the most eccentric flankers in the four-flanker condition only partly fell within the crowding zone (see Figures 2 and 7), would imply that interference is determined by the number of features falling in that region, and not the number of complete flankers. However, when considering possible inward-outward asymmetries in crowding effects, it is possible to argue that it is in fact the number of complete flanking elements that is critical here.

Bouma (1978) was the first to propose that the receptive fields that determine the empirically observed crowding zone are elongated toward the periphery, and therefore to the left in the LVF and to the right in the RVF. More recently, Nandy and Tjan (2012) have proposed a theory of crowding that explains this inward-outward asymmetry. This asymmetry is thought to arise via a modification of the receptive field structure in V1 by the distortion of image statistics caused by saccadic eye movements. Nandy and Tjan argue that the inward-outward asymmetric nature of crowding is a consequence of the properties of lateral connectivity in V1 (isotropic and independent of eccentricity) and the fact that the size of receptive fields of V1 neurons increase linearly with eccentricity (see van den Berg et al., 2010, for an alternative account of inward-outward asymmetry based on the same properties of cortical structure). The two additional ingredients in Nandy and Tjan’s account are the modification of lateral connectivity in V1 at peripheral locations by attention allocated to these locations, and the temporal overlap between the duration that attention is allocated to a particular retinal location and the saccade to that location that is elicited by the attention shift. It is the fact that saccades are generally radial with respect to the fovea that causes the foveal-peripheral anisotropy (inward-outward asymmetry) of crowding. Without going into the finer details of this theory, here we simply examined the extent to which parameters derived from Nandy and Tjan’s theoretical analysis could account for the effects of non-adjacent flankers observed in the present study (see their Figure 3D). Given the letter size (0.6°) and eccentricity (2.7°) used in the present study, Nandy and Tjan’s parameters were found to predict a crowding zone that extended 1.62° inward and 4.32° outward. As shown in Figure 7, this implies that one complete non-adjacent letter falls in the crowding zone. In order to account for the effect of non-adjacent flankers in the present study, it therefore would be the number of complete objects falling in the crowding zone that would determine the amount of interference, rather than the number of visual features. Finally, the model correctly predicts no further decrement in performance when comparing the six-flanker condition with the four-flanker condition tested in Experiment 2.

In support of this general approach, we have shown how applying Nandy and Tjan’s (2012) model of inward-outward asymmetries in crowding can help explain the pattern of serial position effects found for letter-in-string identification in peripheral vision (Chanceaux and Grainger, 2012). However, the obvious critical test of this account of the non-adjacent flanking effects seen in the present study will be to separately examine the effects of outward and inward flankers. This is the object of on-going research.

### Spatial attention and effects of adjacent and non-adjacent flankers

The results of the present study revealed a clear advantage for post-cued letter-in-string identification under unilateral presentation conditions compared with bilateral presentation. We attribute this advantage to the different deployment of spatial attention in these two conditions. In the unilateral condition, a single stimulus in one or the other visual field will attract attention to the stimulus location, hence facilitating processing of the target. In the bilateral condition, attention will remained deployed across visual fields until target location has been identified and attention can be directed to that location. Furthermore, the greater interference seen with different letter flankers compared with homogeneous × flankers, in Experiment 1, can also be explained in terms of differences in the deployment of spatial attention. Homogeneous flankers would facilitate focusing of attention on the target as the odd man out, much like different colored targets were thought to help focus attention in the study by Scolari et al. (2007 see Whitney and Levi, 2011, for a discussion of perceptual grouping, popout, and crowding).

One key result of the present study concerns the different way in which our two attentional manipulations modulated the effects of adjacent vs. non-adjacent flankers. In Experiment 1, spatial attention significantly modulated the effects of non-adjacent flankers (comparing zero- vs. two-flankers) but did not significantly modulate the difference between the two-flanker and four-flanker conditions. The results of Experiment 2 demonstrated that the latter finding was not due to floor effects. The absence of an interaction between number of flankers and unilateral vs. bilateral presentation in Experiment 2, contradicts attentional accounts of such effects (Strasburger et al., 1991), and suggests that different mechanisms underlie the way non-adjacent flankers and spatial attention can affect target identification.

On the other hand, effects of adjacent flankers were significantly reduced under unilateral compared with bilateral presentation, and significantly reduced when flankers were homogeneous Xs compared with different letter flankers. These findings are in line with prior research showing that that attracting attention to the target location can reduce crowding (e.g., Yeshurun and Rashal, 2010). One way to accommodate such attentional influences within an account of crowding as excessive feature integration is via attention boosting the target’s capacity to resist flanker interference (see e.g., the biased competition theory of spatial attention: Desimone and Duncan, 1995). The question then is why the difference between the two-flanker and four-flanker conditions was not affected by the same manipulations of spatial attention? Considering effects of bilateral vs. unilateral presentation, where a possible influence of floor effects was excluded in Experiment 2, one possibility is that while two-flankers still enable focusing of attention on the target location, greater numbers of flankers cause a significant spread of attention away from the target. In this way target identification is still facilitated compared with bilateral presentation conditions, but since flanking stimuli also benefit from allocated attention, flanker interference is not reduced. Our results would therefore reflect the combined influence of crowding and spatial attention on location-specific processing of letter identities (Marzouki et al., 2013).

Finally, no effects of visual field were found in the present study, and no differences in visual field effects were found between unilateral and bilateral presentation. This is in line with prior evidence that visual field differences in letter-in-string identification vary from being weak to absent, and that effects with random letter strings are systematically smaller than those obtained with words (e.g., Bouma, 1973; Jordan et al., 2003). This might well indicate that the very earliest phase of letter string processing involves neural structures (most likely middle occipital gyrus) located in both hemispheres.

### Conclusion

The present study investigated the influence of number of flanking elements and the bilateral vs. unilateral nature of the display in a post-cued letter-in-string identification task. The results revealed a standard crowding effect in both unilateral and bilateral presentation conditions, with target identification being harder when targets were surrounded by two-flankers compared with the no flanker condition. Most important, however, is that a further increase in the number of flankers (from two- to four-flankers) caused a further decrement in performance. This interfering effect of number of flankers was greater for all different letter flankers compared with homogeneous × flankers. Finally, flanker interference was modulated by both the laterality manipulation and the type of flanking stimulus when contrasting zero- and two-flankers, but not when contrasting two- and four-flankers. We argue that the most parsimonious account of the complete set of findings is in terms of flanker interference increasing as more elements enter the crowding zone, plus the way spatial attention, when sufficiently focused, boosts the target’s capacity to limit flanker interference.

## Conflict of Interest Statement

The authors declare that the research was conducted in the absence of any commercial or financial relationships that could be construed as a potential conflict of interest.

## Footnotes

^1^Several studies have also examined effects of number of equidistant flankers using various kinds of stimuli and with varying results (e.g., Wilkinson et al., 1997; Pelli et al., 2004; Põder and Wagemans, 2007; Põder, 2008).

^2^We note that modulation of flanker interference by spatial attention does not contradict accounts of crowding as resulting from pooling of information falling in the crowding zone, in as much as attention can boost the target’s capacity to resist the inhibitory action of flanking stimuli (e.g., Grainger et al., 2010).

^3^Note that this is not the case in studies manipulating the number of equidistant flankers (e.g., Wilkinson et al., 1997; Pelli et al., 2004; Põder and Wagemans, 2007; Põder, 2008).
